# Antioxidant and relaxant activity of fractions of crude methanol extract and essential oil of *Artemisia macrocephala* jacquem

**DOI:** 10.1186/1472-6882-13-96

**Published:** 2013-05-06

**Authors:** Niaz Ali, Ismail Shah, Syed Wadood Ali Shah, Ghayour Ahmed, Mohammad Shoaib, Muhammad Junaid, Waqar Ali, Zahoor Ahmed

**Affiliations:** 1 Department of Pharmacology, Institute of Basic Medical Sciences, Khyber Medical University, Peshawar, Khyber Pakhtunkhwa, Pakistan; 2 Department of Pharmacy, University of Malakand, Chakdara, Dir, Khyber Pakhtunkhwa, Pakistan; 3 Institute of Pharmaceutical Sciences, Kohat University of Science and Technology, Kohat, Khyber Pakhtunkhwa, Pakistan; 4 Pharm-D Scholar, Department of Pharmacy, Abasyn University, Peshawar, KPK, Pakistan; 5 Department of Biochemistry, Khyber Medical College, Peshawar, Khyber Pakhtunkhwa, Pakistan

## Abstract

**Background:**

The current work is an attempt to know about additional chemical profile of *Artemisia macrocephala.* Antioxidant activity is performed as the plant is reported to contain flavonoids, which have antioxidant activity in general. Relaxant activity of fractions of crude methanol extract is performed to know in which fraction(s) the relaxant constituents concentrate as we have already reported that its crude methanol has relaxant activity. Antispasmodic activity of essential oil is also performed as the plant is rich with essential oil.

**Methods:**

Phytochemical profile of the plant is performed. Free radical scavenging activity was performed using 2, 2-diphenyl-1-picrylhydrazyl (DPPH). Relaxation activity tests of fractions and essential oil of *Artemisia macrocephala* were performed on sections of rabbits’ jejunum. Calcium chloride curves were constructed to investigate the mode of action of plant extracts and its essential oil.

**Results:**

We detected carbohydrates, flavonoids and saponins in *A. macrocephala*. At concentration 0.005 mg/ml, free radical scavenging activity of ethyl acetate fraction was 121.5 ± 2.02% of ascorbic acid.

*n*- hexane fraction relaxed spontaneous activity with EC_50_ 0.74 ± 0.04 mg/ml. Essential oil relaxed spontaneous activity with EC_50_ 0.8 ± 0.034 mg/ml. Chloroform and ethylacetate fractions relaxed both spontaneous and KCl-induced contractions suggesting its possible mode through calcium channels. Constructing calcium chloride curves, the test fractions showed a right shift in the EC_50_. Essential oil at concentration 0.1 mg/ml produced right shift with EC_50_ (log [Ca^++^]M) -2.08 ± 0.08 *vs.* control with EC_50_ -2.47 ± 0.07. The curve resembled the curves of verapamil, which caused a right shift at 0.1 μM, with EC_50_ -1.7 ±0.07 *vs.* control EC_50_ (log [Ca^++^]M) -2.45 ± 0.06.

**Conclusions:**

Crude methanol and its fractions (ethyl acetate, chloroform and butanol) are rich sources of antioxidant constituents. The relaxing constituents following calcium channel blocking mechanisms are more concentrated in *n*-hexane, chloroform and ethyl acetate fractions that warrant isolation.

## Background

*Artemisia macrocephala* belongs to family Asteraceae, which is of great medicinal importance. *Artemisia macrocephala* Jacquem (Synonym: *Artemisia griffithiana* Bioss) is 20–30 cm tall. It is called “Tarkha” in Pashto language. It is abundantly found in northern areas of Pakistan. Asteraceae contributes to 10% of flowering plants. That is why it is regarded as the largest group of flowering plants [1]. The Asteraceae contains 1,600 genera and 25,000 accepted species which are of great importance [2]. Mainly, plants of Asteraceae are distributed in the semi-arid regions of tropics and subtropics of the world. So far, biologically active compounds like sesquiterpenes, lactones, volatile oils and terpenoidal compounds have been reported from the plants belonging to Asteraceae, which have anthelmintic, anti-parasitic, cytotoxic, and *in vivo* trypanocidal activities [3,4]. Most of the species of genus Artemisia are used as a food, forage, medicines, soil stabilizers, and for ornamental purposes. Artemisia is the largest genera of the family, which contains more than 400 accepted species that are distributed throughout the world [5]. Some species are allergenic or toxic, while some are invasive weeds [6].

Previously, reported constituents from essential oil of *A. macrocephala* are propionic acid, acetic acid, enanthic acid and isovaleric acid. Its oil also contains camphene, α-pinine, β-pinine, limonene, p-cymene, borneol, 1,8-cineole, and camphor [7]. Upon preliminary phytochemical screening, *A. macrocephala* tested positive for the presence of flavonoids, alkaloids, terpenes and saponins as per our previous report [8].

We were interested in investigating some more groups of phytochemicals of crude methanol extract of *A. macrocephala* (Am. CMeOH) to standardize it. Since the plant was reported to have antispasmodic activity, hence, the current work is a step forward attempt to know in which fractions the relaxant constituents concentrate. Antioxidant activity was also performed as the plant contains flavonoids, which have antioxidant activity in general [9,10].

## Materials and methods

### Collection and authentication of plant’s materials

Fresh aerial parts of young shoots of *A. macrocephala* were collected in the month of August, 2009 from the hills near to Badwan Chowk, Dir Lower, Khyber Pakhtunkhwa, Pakistan. The plant was authenticated by plant taxonomist, Dr. Jehandar Shah, Vice Chancellor, Shaheed Benazir Bhutto University, Dir Upper, Sheringal. A voucher specimen “AM-01-2009” was submitted to the herbarium of Department of Botany, University of Malakand.

### Extraction

The aerial parts of plant were subjected to shade drying at room temperature. The plant’s materials were crushed and then pulverized by mean of a mechanical grinder to obtain fine powder. The fine powder (5.5 kg) was then macerated in commercial grade (50%) methanol with occasional shaking for 22 days at room temperature. The materials were filtered off. The process was repeated 3 times. The filtrates were combined and evaporated under reduced pressure, using a rotary evaporator, till a dark-greenish color extract was obtained. It yielded 555 gram.

### Fractionation

500 grams of crude methanol extract of *A. macrocephala* (Am.CMeOH) were suspended in distilled water (400 ml), which was successively fractionated with 400 ml of *n*-hexane. The process was repeated till complete fractionation. Similar procedure was followed for fractionation with chloroform, ethyl acetate, *n*-butanol till it successively gave 40 g of *n-*hexane fraction (Am. *n-*hex), 61.7 g of chloroformic fraction (Am.Cl), 39.4 g of ethyl acetate (Am.EtOAc), 38.8 g of butanol fraction (Am. BuOH) and 40.7 g of residual aqueous fraction (Am. Aq). The fractions were stored in refrigerator.

### Drugs and chemicals

All tests solutions were prepared in distilled water on the same day of experiments. Rabbits of either sex were housed in the animal house of University of Malakand. Ethical committee of the department of pharmacy approved the study protocols (Case approval # E- AM-01-2009) as per “Bye Laws 2008 of the University of Malakand (Scientific procedures Issue- I)”.

### Statistical analysis and calculations

Microsoft Excel sheet was used to calculate means and standard deviations. Bar charts for antioxidant activity were drawn in Excel sheet. Graph pad prism was used to draw the curves for determination of EC_50_. The level of significance was tested at P ≤ 0.05 using students “t” test.

### Phytochemical profile and essential oil

We have already reported that the plant contains alkaloids, flavonoids, saponins and terpenes [8]. Some more phytochemical profile was investigated for the presence of carbohydrates, anthraquinone glycosides and tannins as per reported procedures [11]. Essential oil, from fresh aerial parts, was obtained by simple steam distillation method which was subjected to GC/MS analysis.

### Antioxidant activity

The antioxidant potential of crude methanol extract and its fractions was determined using DPPH free radical scavenging activity. Test samples of Am.CMeOH, Am. *n*-hex, Am. Cl, Am. EtOAc, Am.BuOH and Am.Aq (2 ml each) of several concentrations (0.5 mg/ml, 0.2 mg/ml, 0.1 mg/ml, 0.05 mg/ml and 0.005 mg/ml) were prepared. 2 ml of DPPH (2%) was added to all test samples. The test solutions and DPPH was prepared in methanol. Ascorbic acid was used as a standard. All the experiments were performed in triplicates. Absorbance of the sample was noted at 517 nm using methanol as blank [12]. Radical scavenging activity of all test samples was plotted as % of ascorbic acid at respective concentrations.

### Effects of various fractions on spontaneous rabbits’ jejunum preparations and potassium chloride induced contractions

The antispasmodic activity of various fractions of Am.CMeOH was performed on spontaneous rabbits’ jejunum preparations as per standard protocols [13-17]. After cervical dislocation, rabbits’ abdomens were opened. About 1.5 cm sections of jejunum were separated and placed in petri dishes containing Tyrode’s solution. The tissues were constantly aerated with carbogen gas. These tissues were then mounted in organ bath containing Tyrode’s solution. Temperature was maintained at 37 ± 1°C. After stabilization of the tissues for about 30 minutes, fractions of Am.CMeOH were tested in 0.01, 0.03, 0.1, 0.3, 1.0, 3.0, 5.0 and 10.0 mg/mL concentrations. We also screened its essential oils for possible relaxant effects. The experiments were performed six times. For understanding of possible mechanisms of action, the test fractions were also tested on (80 mM) KCl-induced contractions in rabbits’ jejunum preparations [18].

### Effects on voltage gated calcium channels

Since the test fractions and essential oil relaxed high KCl-induced contractions, hence, we constructed calcium chloride curves as relaxing effect on KCl-induced contractions are usually (not necessarily) following voltage gated channels. The tissues were treated with different concentrations of *A. macrocephala* in decalcifying medium. An incubation time of 1 hr was given. Briefly describing, we exposed the tissues to calcium-free Tyrode’s solution having EDTA (0.1 mM). Then, we performed experiments in potassium-rich Tyrode’s solution. Earlier, the tissues were stabilized for about 30 minutes in normal Tyrode’s solution. Concentration response curves for calcium were constructed in decalcified tissues in cumulative manner at concentrations 1 × 10^−4^ - 256 × 10^−4^ M [8,14-17].

## Results and discussion

**Figure 1:**
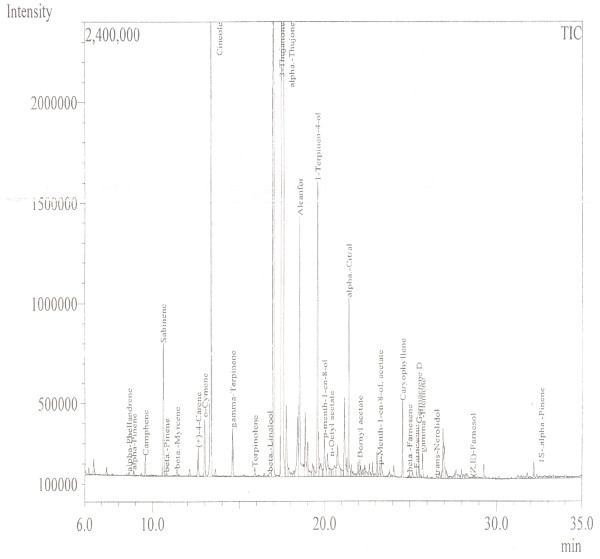
**GC/MS Spectrum of essential oil of *Artemisia macrocephala*.
**

**Figure 2:**
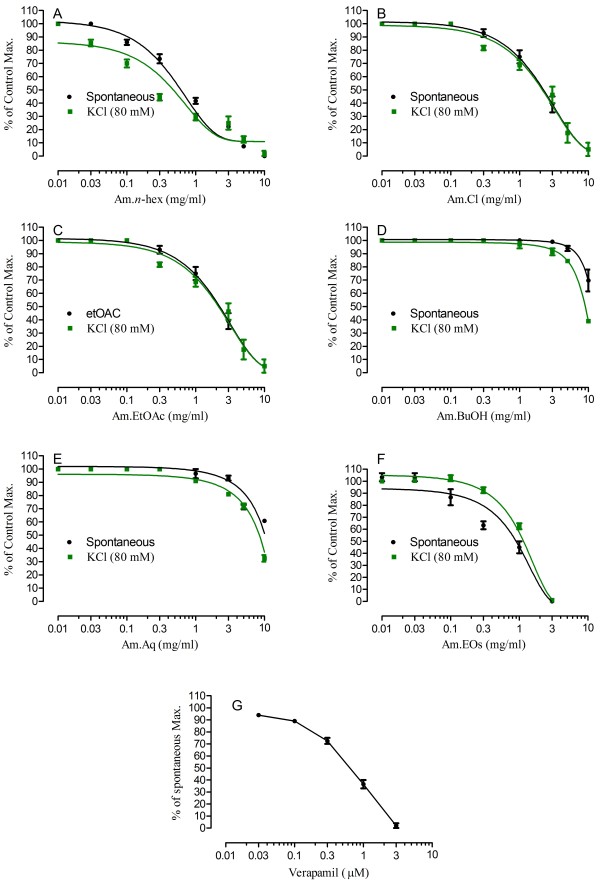
Effects of various fractions of Artemisia macrocephala and verapamil on spontaneous and KCl -induced contractions in rabbits’ jejunum preparations.

**Figure 3:**
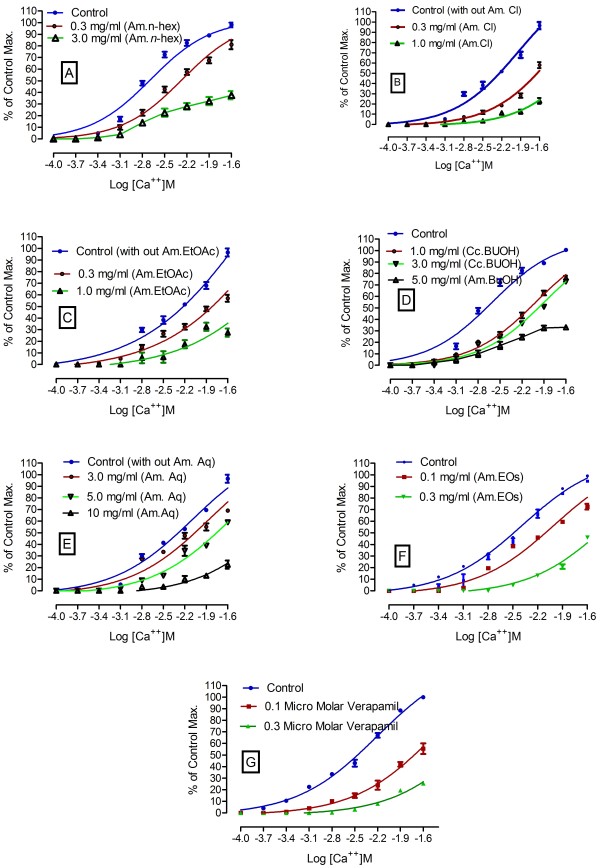
**Effects of various fractions of methanol extract of *Artemisia macrocephala *and verapamil on calcium chloride curves.**

**Figure 4:**
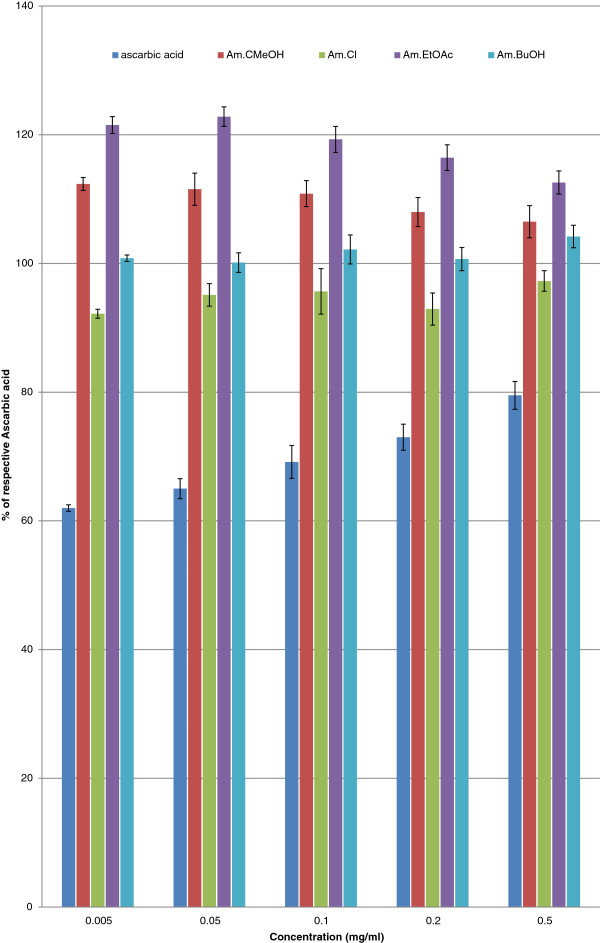
**Antioxidant activity of crude methanol extract of *Artemisia macrocephala *as % of ascorbic acid.**

Phytochemical analysis of *A. macrocephala* confirmed the presence of carbohydrates, flavonoids, alkaloids and saponins. It tested negative for proteins, tannins and glycosides. The GC/MS spectrum is presented in Figure 1. Alpha Thujone (56.24%), 3-Thujanone (11.73%), cineol (10.76%), in addition to previously reported constituents, were the most prominent constituents of the essential oil of *A. macrocephala.* It has been also reported that flavonoids, in general, have antioxidant, cytotoxic, anti-tumor and antispasmodic activity [19,20]. Hence, it was deduced that flavonoids may be responsible for relaxant and antioxidant activities. As crude methanol extract of *A. macrocephala* has been reported for antispasmodic activity, hence, we currently fractionated the methanol extract to know in which fraction(s) the relaxant constituent(s) concentrate. Figure 2 describes the effects of various fractions of crude methanol extract of *A. macrocephala* on spontaneous rabbits’ jejunum preparations and on potassium chloride induced contractions. EC_50_ values for Am. *n-*hex are 0.74 ± 0.04 and 0.5 ± 0.03 mg/ml, respectively, for spontaneous and KCl-induced contractions (Figure 2A). EC_50_ values for Am.Cl and Am.EtOAc on spontaneous contractions are 2.0 ± 0.1 and 2.3 ± 0.1 mg/ml (Figure 2B and Figure 2C). EC_50_ values for effects on KCl-induced contractions are 2.0 ± 0.1 and 2.2 ± 0.1 mg/ml (Figure 2D and Figure 2E). While EC_50_ for effects of essential oil of Artemisia on spontaneous and KCl-induced contractions are 0.8 ± 0.3 and 1.1 ± 0.1 mg/ml. Figure 2G describes the effects of verapamil on spontaneous rabbits’ jejunum preparations with EC _50_ 0.57 ± 0.04 μM. It is important to mention that *n-*hexane, chloroform, ethylacetate and essential oil relaxed both the spontaneous and KCl-induced contractions up to 100%. Therefore, these fractions can be sources of calcium antagonists. Calcium chloride curves (CRCs) were constructed to investigate about its possible mechanism through calcium channels or otherwise (Figure 3). Our main targets were *n-*hexane, chloroform, ethylacetate and essential oil because of their relaxing effects on spontaneous and KCl-induced contractions. Curves were also drawn for Am. BuOH and Am. Aq fractions because of their relaxing effects at high concentrations. According to Figure 3A, *n-*hexane fraction at concentration 0.3 mg/ml produced a right shift EC_50_ -2.3 ± 0.1 *vs.* control EC_50_ -2.6 ± 0.09. In the presence of 0.3 mg/ml of Am. EtOAc, EC_50_ is −1.62 ± 0.04 *vs.* control −2.2 ± 0.2. Am.Cl at concentration 0.3 mg/ml produced a right shift with EC_50_ -1.82 ± 0.1. Am. BuOH and Am.Aq produced rightward shift at respective high concentrations i.e. 5.0 and 10 mg/ml. Essential oil at concentration 0.1 mg/ml produced right shift (Figure 3F) with EC_50_ -2.08 ± 0.08 *vs.* control EC_50_ -2.47 ±0.07. The curves resembled the curves of verapamil which produced a right shift at 0.1 μM with EC_50_ -1.7 ±0.07 *vs.* control −2.45 ± 0.06 (Figure 3G). It is the free calcium levels that are responsible for contractile effects of jejunum. The increase in cellular calcium levels is either by the release of calcium from stores in sarcoplasmic reticulum or is through the influx of calcium passing through L type voltage gated calcium channels into the cells [21]. Intracellular and extracellular calcium stores also exchange with one other that are responsible for the periodic depolarization and repolarization of jejunal tissues [22,23]. Since it is not necessary that the relaxing effects on KCl-induced contraction always follow voltage gated channels, therefore, we constructed calcium chloride curves for fractions which had relaxing effects on the KCl-induced contractions. All the fractions showed right shift in the EC_50._ Hence, it is suggested that these fractions are sources of relaxing constituents. The order of potency based on the right shift is: 0.1 mg/ml Am.EOs > 0.3 mg/ml Am. *n-*hex=0.3 mg/ml Am.Cl. It suggested that the relaxing constituents are concentrated in these fractions that inhibited the flow of calcium through voltage gated channels [13,15]. The right shift may be attributed to the presence of phytochemicals like flavonoids and saponins as these types of compounds, in medicinal plants, have been reported to have calcium channel blocking activity [24]. Free radicals are extremely dangerous as they are very unstable and damage the cells. Free radicals like superoxide radical (O2•-), hydroxyl radical (HO•), nitric oxide radical (NO•) and the lipid peroxyl radical (LOO•) damage the cells. Compounds that have lone pair of electrons can simply stabilize these free radicals and thus they are protective in nature. It has been proved that DPPH method is rapid, simple, accurate and inexpensive. It measures the ability of different compounds to act as free radical scavengers or hydrogen donors that helps to evaluate the antioxidant activity of various foods and beverages. Results of antioxidant activity are shown in Table 1. It is evident that Am.CMeOH, Am.Cl, Am.EtOAc and Am.BuOH showed excellent DPPH free radical scavenging activity. Their efficacy is comparable with ascorbic acid at respective test concentrations (Table 1). When plotted the results as % of ascorbic acid activity (Figure 4), it is evident that the antioxidant constituents are more concentrated in ethyl acetate fraction. At 0.005 mg/ml, its free radical scavenging activity is 121.5 ± 2.02% (n=3) of ascorbic acid. It is evident from Table 1 that ethyl acetate, chloroform and butanol fractions are good sources of antioxidant constituents as well.

**Table 1:** **Results of antioxidant activity of crude methanol extract and fractions of *Artemisia macrocephala***

**Concentration (mg/ml)**	**Test samples**						**Standard**
	**Am.CMeOH**	**Am. *n*-hex**	**Am. Cl**	**Am. EtOAc**	**Am. BuOH**	**Am.Aq**	**Ascorbic acid**
0.005	69.66 ± 1.52	16.83 ± 2.84	57.16 ± 2.56	75.33± 2.02	62.50 ± 2.17	28.33 ± 1.75	62.00 ± 0.5
0.05	72.50 ± 2.50	22.66 ± 2.51	61.83 ± 2.02	79.83 ± 2.25	65.09 ± 2.50	32.16 ± 2.56	65.33 ± 1.0
0.1	76.66 ± 1.75	26.33 ± 1.25	66.16 ± 3.54	82.50 ± 2.50	70.66 ± 1.60	34.66 ± 2.02	69.16 ± 0.7
0.2	78.83 ± 1.52	31.50 ± 1.50	67.83 ± 2.02	85.00 ± 2.0	73.50 ± 1.80	36.66 ± 5.20	73.00 ± 1.3
0.5	84.66 ± 1.52	38.66 ± 1.25	77.33 ± 2.25	89.50 ± 1.80	82.83 ± 1.75	42.6 ± 2.75	79.50 ± 0.5

## Conclusions

Crude methanol and its fractions (ethyl acetate, chloroform and butanol) are rich sources of antioxidant constituents. The relaxing constituents following calcium channel blocking mechanisms are more concentrated in *n*-hexane, chloroform and ethyl acetate fractions that warrant for its isolation.

## Limitations of the study

The antioxidant activity requires further work in some other antioxidant models including superoxide, hydroxyl and nitric oxide free radicals to improve the quality of work.

## Abbreviations

(Am.CMeOH): Crude methanol extract of *Artemisia macrocephala*; (Am. n-hex): *n*-hexane fraction of *Artemisia macrocephala*; (Am. Cl): Chloroformic fraction of *Artemisia macrocephala*; (Am. EtOAc): Ethyl acetate fraction of *Artemisia macrocephala*; (Am. BuOH): Butanol fraction of *Artemisia macrocephala*; (Am.Aq): Residual aqueous fraction of *Artemisia macrocephala.*

## Competing interests

The authors have no competing interests.

## Authors’ contributions

NA participated in collection, interpretation of data; and writing and major revision of the manuscript. IS (M. Phil Research Scholar) assisted in collection and tabulation of data. SWA assisted data collection and literature survey. GA assisted in data collection and interpretation of data. SM helped in literature survey. MJ assisted in literature survey and phytochemistry. WA assisted in literature survey. ZA helped determining the phytochemistry and secondary metabolites of the test samples. All authors read and approved the final version of the manuscript.

## Pre-publication history

The pre-publication history for this paper can be accessed here:

http://www.biomedcentral.com/1472-6882/13/96/prepub
